# A Discovery of a Genetic Mutation Causing Reduction of Atrogin-1 Expression in Broiler Chicken Muscle

**DOI:** 10.3389/fgene.2019.00716

**Published:** 2019-08-15

**Authors:** Jinxiu Li, Yiqing Hu, Li Li, Yuzhe Wang, Qinghe Li, Chungang Feng, He Lan, Xiaorong Gu, Yiqiang Zhao, Mårten Larsson, Xiaoxiang Hu, Ning Li

**Affiliations:** ^1^State Key Laboratories of Agro-biotechnology, College of Biological Science, China Agricultural University, Beijing, China; ^2^CAS Key Laboratory of Genome Sciences and Information, Beijing Institute of Genomics, Chinese Academy of Sciences, Beijing, China; ^3^Science for Life Laboratory, Department of Medical Biochemistry and Microbiology, Uppsala University, Uppsala, Sweden; ^4^National Engineering Laboratory for Animal Breeding, China Agricultural University, Beijing, China; ^5^College of Animal Science and Technology, Yunnan Agricultural University, Kunming, China

**Keywords:** Atrogin-1, E3 ubiquitin ligase, chicken, SNP, muscle development

## Abstract

Chickens are bred all over the world and have significant economic value as one of the major agricultural animals. The growth rate of commercial broiler chickens is several times higher than its Red Jungle fowl (RJF) ancestor. To further improve the meat production of commercial chickens, it is quite important to decipher the genetic mechanism of chicken growth traits. In this study, we found that broiler chickens exhibited lower levels of E3 ubiquitin ligase muscle atrophy F-box (MAFbx or Atrogin-1) relative to its RJF ancestor. As a ubiquitin ligase, Atrogin-1 plays a crucial role in muscle development in which its up-regulation often indicates the activation of muscle atrophic pathways. Here, we showed that the Atrogin-1 expression variance partly affects chicken muscle growth rates among different breeds. Furthermore, we demonstrated that the reduced expression of Atrogin-1 in broiler chickens was ascribed to a single nucleotide polymorphism (SNP), which inhibited the binding of transcription regulators and attenuated the enhancer activity. The decreased Atrogin-1 in broiler chickens suppresses the catabolism of muscle protein and preserves muscle mass. Our study facilitates the understanding of the molecular mechanism of chicken muscle development and has a high translational impact in chicken breeding.

## Introduction

It has been proven that Red Jungle fowl (RJF) (*Gallus gallus*) is a major ancestral contributor of domestic chickens which led to a wide spectrum of breeds (Liu et al., 2006). During the process of human selection and breeding, chicken phenotypes vary among different breeds. Accumulation of favorable mutations is associated with enhanced growth, reproduction and disease resistance, etc. (Zhou and Lamont, 1999; Wright et al., 2006; Wang et al., 2014). Commercial chicken lines were mainly selected for the production of meat and eggs. The growth rate of ordinary domesticated chicken breeds is about six to eight times higher than that of wild chickens, which has further been improved to 5 to 10 times in commercial broilers (Zuidhof et al., 2014). Profound differences in muscle growth and development exist between domesticated and wild chickens. Thus, deciphering the genetic basis of these differences is of high importance to both biological science and the agricultural economy.

Dissection of the genetic mechanisms of growth traits in livestock could greatly advance our understanding in animal breeding. Thus, genetic factors affecting chicken growth traits have been widely studied over the last two decades. There are more than 3,700 quantitative trait loci (QTL) associated with growth traits based on the chicken QTL database. Most QTLs are located on macrochromosomes including chicken (*Gallus gallus*) chromosome 1, 2 and 4 (Deeb and Lamont, 2003; Kerje et al., 2003; Atzmon et al., 2006; Xie et al., 2012). Rubin et al. screened “selective sweeps” in commercial broilers and showed that insulin-like growth factor 1 (IGF1) and pro-melanin-concentrating hormone (PMCH) might serve as surrogates in the selection of muscle growth (Rubin et al., 2010). Other studies have shown that Thyroid Hormone Responsive Spot14 (THRSP) (d’Andre Hirwa et al., 2010), FOXO1a and KPNA3 (Xie et al., 2012) might also be related to chicken growth traits.

In this study, our analysis of the thigh muscle tissue transcriptomes of 7-day-old Arbor Acres (AA, a commercial broiler chicken line which is a Cornish cross) has revealed lower expression of E3 ubiquitin ligase muscle atrophy F-box (MAFbx or Atrogin-1) compared with RJF. Given the crucial role of Atrogin-1 in the degradation of myogenic factors, we speculate that the differential expression pattern of this gene might in part account for the skeletal muscle mass in the AA broiler.

The size of myofibers is determined by the dynamic balance between protein synthesis and degradation (Sandri, 2008; Bonaldo and Sandri, 2013). There are three cellular mechanisms of protein degradation: the ubiquitin-proteasome pathway, lysosomes, and mitochondrial proteases (Goldberg, 2003; Ciechanover, 2005; Mizushima et al., 2008). The ubiquitin-proteasome system (UPS) is an ATP-dependent, highly specific protein degradation system in eukaryotes, which accounts for more than 80% of the protein degradation, playing a crucial role in regulating cellular protein metabolism (Hershko and Ciechanover, 1992; Hershko and Ciechanover, 1998; Rose, 2005).

Ubiquitin ligases MAFbx/Atrogin-1 and MuRF1 were identified in 2001 by the laboratories of Glass and Goldberg (Bodine et al., 2001; Gomes et al., 2001). Previous studies have shown the crucial role of the ubiquitin–proteasome system (UPS) in muscle wasting through the degradation of myofibrillar proteins (Attaix et al., 2005; Jung et al., 2009). These two E3 ligases are critical components of the UPS and have been widely studied in muscle atrophy (Sacheck et al., 2004; Foletta et al., 2011). Muscle atrophy F-box protein (MAFbx or Atrogin-1) regulates the degradation of various structural and regulatory factors of the muscle (Bodine et al., 2001; Gomes et al., 2001). Previous studies have shown that myotubes over-expressing Atrogin-1 reduced the myofibrillar diameter, which is indicative of muscle atrophy *in vitro*. On the contrary, denervation did not induce a reduction of muscle mass in Atrogin-1 knockout mice (Bodine et al., 2001). Moreover, Atrogin-1 targets elongation initiation factor 3 subunit 5 (EIF3-F) and the myoblast determination protein (MyoD), which regulates protein synthesis and muscle differentiation (Li et al., 2004; Tintignac et al., 2005; Lagirand-Cantaloube et al., 2008). Thus, Atrogin-1 plays an important role in the regulation of muscle homeostasis and development.

There is also evidence showing an important role of Atrogin-1 in chicken skeletal muscle development (Nakashima and Ishida, 2015). Our laboratory has demonstrated that the expression of Atrogin-1 was increased robustly in fasting chickens, whereas this elevation was reversed by re-feeding (Li et al., 2011). It has also been reported that the mRNA level of Atrogin-1 in skeletal muscle was lower in broiler chicks than in layer chicks (Nakashima et al., 2009; Ohtsuka et al., 2011). It is thought that the higher growth rate observed in broiler chicken could be attributed to the disparity in expression of UPS-related genes and subsequent perturbation to the proteolytic system in the skeletal muscle (Saneyasu et al., 2015). In addition, Li et al. also demonstrated that a panel of ubiquitin-related genes were differentially expressed in RJF and avian broiler chickens (Li et al., 2012).

According to our previous RNA-seq results, we found that the expression of Atrogin-1 was decreased in the skeletal muscle of broiler chickens compared to wild RJF, suggesting that Atrogin-1 might determine the disparity in muscle mass among the breeds. Here, we attempt to decipher the genetic mechanism underlying the differential expression of Atrogin-1 among chicken breeds. In this study, we identified a causal single nucleotide polymorphism (SNP) of Atrogin-1 in broiler chickens at the genetic level using large-scale PCR combined with the Ion torrent sequencing technique, and systematically studied the mechanism underlying the differential gene expression. We aimed to unveil the pattern of the variation in Atrogin-1 caused by the artificial selection of broilers. This study will help illustrate the molecular mechanism of muscle development to apply it to chicken breeding.

## Material and Methods

### Animals

Commercial broiler chickens (AA), Cobbs, the Chinese Dehong (RJF), Yunnan local variety Chahua (CH), Tibetan chickens, Chinese domestic Mini chickens, Silkies, Fast Silkies and Leghorns were used in this study. The Chinese Dehong is a strain of RJF from South China and was obtained from the Wild Animal Rescue Shelter Center of the Yunnan province. The AA broiler chickens were bought from Beijing Huadu Company. Cobb chickens were bought from the Beijing broiler chicken company. CH, Tibetan chickens, Mini chickens, Silkies and Fast Silkies were acquired from the Jiangsu Institute of Poultry Science. The Leghorns were provided by the China Agricultural University. After hatching, the chickens were fed the same diet and sacrificed at 7 days old according to local standards of animal welfare. The animal welfare committee of the State Key Laboratory approved all animal care and experimental procedures for Agro-biotechnology of China Agricultural University with approval number SKLAB-2012-3. Other DNA samples used for sequence analysis were from the Jiangsu Institute of Poultry Science.

### Expression Analysis

Thigh muscle tissues from 7-day-old chickens were snap frozen in liquid nitrogen and then stored at -80°C. Total RNA was extracted using TRIzol Reagent (Invitrogen) according to the manufacturer’s instructions. The RNA preparations were treated with RNase free DNase I to remove potentially contaminating DNA.

RNA was reverse-transcribed into first-strand cDNA with Moloney Murine Leukemia Virus transcriptase (Promega) and oligo (dT) (TaKaRa Bio Inc., Shiga, Japan) using 2 μg of total RNA. The expression of the specific gene was quantified by real-time PCR using a Roche LightCycler480 instrument with the SYBR Green I Master Mix (Roche Applied Science, Indianapolis City, IN, USA). For RT PCR amplification, cDNA was pre-denatured at 95°C for 10 min followed by 40 cycles of 95°C for 30 s, and 60°C for 1 min. The relative expression level of the target gene was normalized to that of the housekeeping gene GAPDH (glyceraldehyde 3-phosphate dehydrogenase) by 2^-ΔΔCT^.

### Antibody and Western Blot

Protein was extracted from frozen tissues with Cell Lysis Buffer (Beyotime). The proteins (40 µg of total cell protein per lane) were separated by 10% SDS-PAGE and transferred to polyvinylidene difluoride (PVDF) membranes according to standard protocols. The membranes were blocked and incubated with anti-Atrogin-1 (1:500, sc-27644, Santa Cruz), anti-Akt (1:2,000, 9272, Cell Signaling), anti-Phoshpo-Akt (Ser473) (1:1,000, 4060, Cell Signaling) antibodies, anti-FOXO3A (1:1,000, ab111993, Abcam), anti-Phoshpo-FOXO3A (1:1,000, ab26649, Abcam), anti-FOXO4 (1:1,000, ab24505, Abcam), anti-mTOR (1:1,000, 2983S, CST), anti-Phoshpo-mTOR (S2448) (1:1,000, ab109268, Abcam), anti-smad2 (1:1,000, 5339S, CST) or anti-smad3 (1:1,000, RLT4333, Ruiyingbio) antibodies overnight at 4°C. The membranes were subsequently incubated with horseradish peroxidase (HRP) conjugated goat anti-mouse or goat anti-rabbit secondary antibodies (1:10,000) and visualized using SuperSignal West Dura Extended Duration Substrate (Thermo Scientific).

### Ion Torrent Sequence of the Atrogin-1 Gene in Eight Different Breeds

Eight breeds (RJF, CH, Tibetan chicken, Mini chicken, Fast Silkie, Silkie, Cobb and AA) were used for sequencing and to generate mutation information around the Atrogin-1 gene region. Each breed had 50 samples and the DNA samples of each breed were diluted into the same concentration and mixed by equal volume.

A 40 kb (chr 2: 143,690,694-143,730,638 bp in galGal3 reference genome) region was separated into 16 different segments. Primers were designed for overlapping fragments (primers list see **Supplementary Table 1**). The template of each breed was amplified by KOD DNA polymerase (Toyobo) and purified with electrophoretic separation on a 1.5% agarose gel and a QIAquick Gel Extraction Kit (Qiagen). Subsequently, the purified DNA products of each breed were pooled by equal mole and sequenced using an Ion Torrent PGM system. Different breeds were separated by different barcodes.

The DNA were sonicated with the BioRuptor UCD-200 TS Sonication System. The fragmented DNA was end-repaired and purified to prepare for ligation to Ion adapters. The library was size selected with the E-Gel SizeSelect Agarose Gel. The DNA concentration of the amplified sequence library was estimated through Qubit2.0 (Qubit dsDNA BR Assay Kit, Thermo Scientific).

Emulsion PCR was carried out using the Ion OneTouch 200 Template Kit v2 DL (Life Technologies) according to the manufacturer’s instructions. Sequencing of the amplicon libraries was carried out on a 314-chip using the Ion Torrent PGM system and by employing the Ion Sequencing 200 kit (Life Technologies) according to the supplier’s instructions. After sequencing, the individual sequence reads were filtered by the PGM software to remove low quality and polyclonal sequences. Sequences matching the PGM 3′ adaptor were also automatically trimmed. All PGM quality-approved, trimmed and filtered data were exported as sff files. The sequencing data of each chicken line were submitted to SRA with the accession number PRJNA530978.

### SNP Detection and Analysis

Ion torrent sequence data was analyzed with Torrent Suit software. The genome sequence of chr 2: 143,690,694-143,730,638 (galGal3 version), downloaded from UCSC, was used as reference. A torrent variant caller was used to obtain SNPs information. Variation frequency is the ratio of mutant reads to total reads for each SNP. The mutant site with a variation frequency above 20% is considered a polymorphism site. Allele counts at identified SNP positions were used to identify signatures of selection in sliding 500-bp windows with a step size of 250 bp for each breed. For each window, the heterozygosity (*Hp*) value was calculated using the formula ***H***_p_ = 2Σ***n***_MAJ_Σ***n***_MIN_/(Σn_MAJ_ + Σ***n***_MIN_)^2^. Σ***n***_MAJ_ is the sum of the major allele reads, and Σ***n***_MIN_ is the sum of the minor allele reads for all significant SNPs in one window.

Re-sequencing data of High Quality chicken Line A (HQLA) and native LiYang (LY) chickens were analyzed with GATK best practices (https://software.broadinstitute.org/gatk/best-practices/). Prior mapping, adapter sequences were deleted and then the reads that contained more than 50% low quality bases, or more than 5% N bases, were removed. Qualified reads were aligned to the chicken reference genome with BWA-MEM (version 0.7.10) using ‘-t 10 -M’ as parameters. Initial BAM files were further processed with reordering, sorting and duplicate marking, utilizing the Picard (picard-tools-1.56) package followed by base quality recalibration using the BaseRecalibrator tools in the Genome Analysis Toolkit (GenomeAnalysisTK-3.7, GATK). Raw variants were called for individual bases using HaplotypeCaller. The gvcf files that were generated from HaplotypeCaller were subjected to joint variant calling using Genotype GVCFs. The VariantFiltration command was employed to exclude potential false-positive variant calls. Subsequently credible SNPs were identified after using strict filtering criteria with parameters: minor allele frequency <0.05 and a call rate >90% in each population.

### Cell Culture

DF1 (a chicken fibroblast cell line) cells were cultured at 37°C in a 5% CO_2_ atmosphere in Dulbecco’s Modified Eagle’s Medium (DMEM) containing 4.5 g/l of glucose (Gibco) and supplemented with 10% fetal bovine serum (FBS).

### Luciferase Reporter Assay

Eight different candidate mutations were selected to perform the luciferase report assays. Wild type and mutant type sequences were generated with PCR and cloned into the pGL3 promoter vector (Promega) to test enhancer activity (see **Supplementary Table 2** for primer information). Because Chr2: 143,723,139 is located in the upstream 2.8 kb region of the Atrogin-1 TSS, we also tested the promoter activity of this site. Therefore, the wild type and mutant type sequence of Chr2: 143,723,139 were also cloned into the pGL3 basic vector (Promega).

The PCR products were around 200 bp in length. *SacI* and *NheI* sites were selected to construct the vector. DF1 cells plated in 24 wells were transfected at 70–80% confluence with 720 ng of the pGL3 reporter plasmid and 80 ng of the pRL-TK Renilla luciferase construct by 2 µl Lipofectamine 2000 (Invitrogen) for each well. The luciferase activity was measured 23–24 h after transfection using the Dual-Glo Luciferase Assay System (Promega) and an Infinite F200 Luminometer (Tecan, Switzerland). Ratios of firefly luminescence/Renilla luminescence were calculated and normalized to the control samples (Basic vector). For each test construct, one expression value was the average of three technical replicates in each plate and three separate operations were carried out to represent the final value. The pGL3 Basic vector, pGL3 Promoter vector and pGL3 Control vector were used as controls.

### Electrophoretic Mobility Shift Assays (EMSAs)

DNA-binding proteins were extracted from DF1 cells with a Nuclear and Cytoplasmic Protein Extraction Kit (Beyotime). The following two (wild-G/mutant-A) 25 bp oligonucleotides were used: 5′- CAGTTAAAACAC (G/A) TCAGGCTGCAGG-3′. EMSAs were performed with 40 fm biotin-labelled, double-stranded oligonucleotide, 10 µg nuclear extract and 1 µg poly (dI-dC), 1 μl 50% Glycerol, 1 μl 1% NP-40, 1 μl 100 mm MgCl_2_ and 2 μl 10× Binding buffer in a total volume of 20 μl reaction system (all the components were from the LightShift Chemiluminescent EMSA Kit, 21048, Thermo Scientific). For competition assays a 100 and 200-fold molar excess of unlabeled double-stranded oligonucleotide was added. Reactions were incubated for 20 min before a biotin-labeled double-stranded oligonucleotide was added. Binding was allowed to proceed for 20 min at room temperature. Five microliters of 5× Loading Buffer was added to each 20 μl binding reaction and pipetted up and down several times to mix. DNA-protein complexes were loaded on a 6.5% native polyacrylamide gel run in 0.5× TBE at 4°C at 100 V until the bromophenol blue dye had migrated approximately 2/3 to 3/4 down the length of the gel. Then the binding reaction was transferred to a nylon membrane according to the kit protocol and subsequently the transferred DNA was crosslinked to the membrane at 120 mJ/cm^2^  for 60 s. The membranes were blocked and incubated with stabilized streptavidin-horseradish peroxidase conjugate and visualized using substrate solution.

## Results

### Differential Expression of Atrogin-1 in Various Chicken Breeds

**Figure 1:**
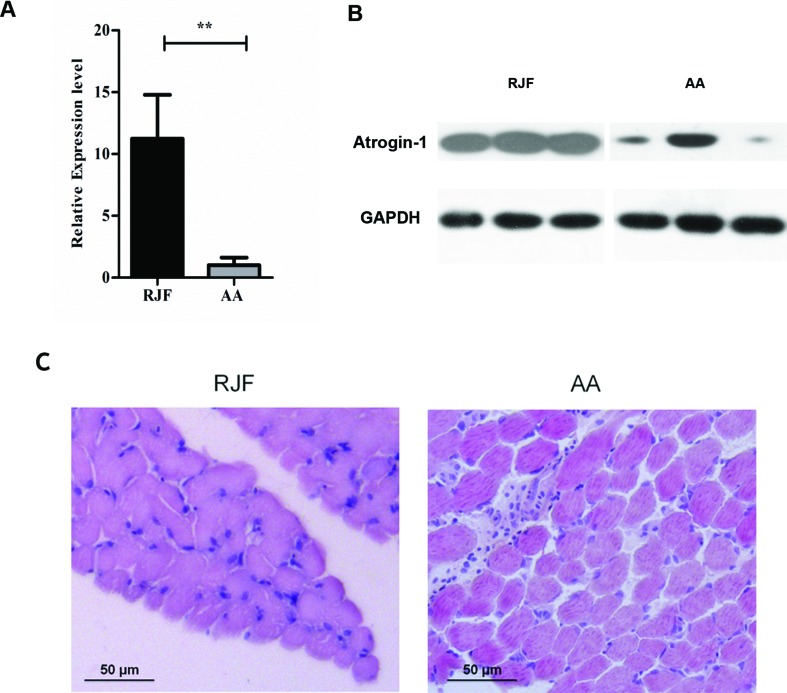
Gene expression level of Atrogin-1 in RJF and AA detected by q-PCR and Western blot **(A)** Relative expression level of *Atrogin-1* detected by q-PCR. The mRNA expression was compared with the *GAPDH* gene. Mean ± SD was present for each group (*n* = 3). The bar means SD of the three sample expressions. *T*-test was used to analyze the significance of different species. ** means *P* < 0.01. **(B)** Protein expression level of Atrogin-1 analyzed by Western blot. Three individuals are shown in three lanes for each group. GAPDH was used as a loading control. RJF (Red Jungle Fowl) and AA (Arbor Acres). **(C)** HE staining of skeletal muscle from 7-day-old RJF and AA chickens (20×).

We first verified the RNA-seq data using Real time PCR and Western Blot and confirmed that the mRNA level of Atrogin-1 in AA was significantly lower than that of RJF (**Figures 1A, B**).

During the process of animal breeding, divergences were observed between commercial chickens and RJF. Specialized commercial broilers have been developed in the past decades characterized by a rapid increase in body weight and muscle mass, etc. Histologic data of 7-day-old chickens indicated that the thigh muscle fibers of AA were significantly thicker than their RJF counterparts (**Figure 1C**). Thus, it is conceivable to speculate that lower levels of Atrogin-1 in AA could be associated with lower muscle catabolism and more muscle mass. Subsequently, we also performed an expression analysis of Atrogin-1 in the skeletal muscle of RJF, CH, AA, Leghorns and Tibetan chickens. Among the five different breeds, the expression of Atrogin-1 in RJF and AA was the highest and lowest, respectively. Besides that, the expression of Atrogin-1 in CH chickens was also higher than in AA broiler chickens (**Supplementary Figure 1**). CH chickens are a local chicken breed from the Yunnan province of China that is closely related to RJF.

### Expression Analysis of Atrogin-1 in Chickens During Different Developmental Stages

**Figure 2:**
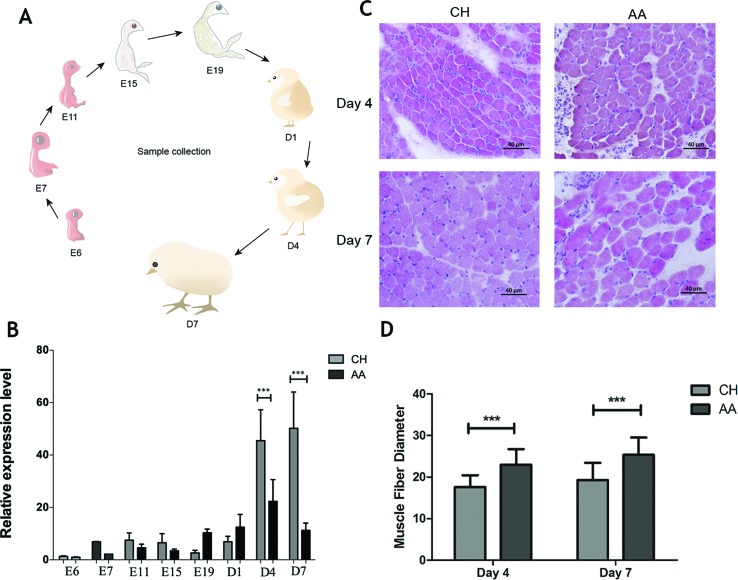
Expression analysis of Atrogin-1 in AA and CH during different developmental stages. **(A)** The developmental stages analyzed. **(B)** Relative expression level of Atrogin-1 detected by q-PCR. The mRNA expression was compared with the GAPDH gene. Mean ± SD was present for each group (n = 3). The bar means SD of the three sample expressions. *** means P < 0.001. Chahua (CH) and Arbor Acres (AA). **(C)** HE staining of skeletal muscle from 4- and 7-day-old CH and AA, upper left is CH at day four, upper right is AA at day four, below left is CH at day seven, below right is AA at day seven. **(D)** Myofiber size of 4- and 7-day- old CH and AA chickens. Three replicates were considered for each species, and 300 myofibers were calculated in each section. Mean ± SD was present for each group (*n* = 3). The bar means SD of the three sample expressions. *** means *P* < 0.001. Chahua (CH) and Arbor Acres (AA).

The expression of Atrogin-1 is known to be induced by fasting, disuse, cancer and other systematic diseases. Our prior study also proved that the expression of Atrogin-1 in chicken skeletal muscles could be enhanced by fasting. To further analyze the expression pattern of Atrogin-1 in different chicken lines, the transcript level of Atrogin-1 in the muscle of CH and AA chickens was measured from various developmental (E6, E7, E11, E15 and E19) and postnatal stages (D1, D4 and D7) (**Figure 2A**). At day four, the Atrogin-1 level was increased significantly in CH chickens whereas no changes were observed in AA chickens (**Figure 2B**), suggesting higher muscle catabolism in CH chickens than in AA chickens. To further validate the hypothesis that the expression of Atrogin-1 would determine the disparity in muscle mass among the breeds, we also performed H&E staining for the muscle tissues of CH and AA chickens obtained from D4 and D7 after birth. Muscle fibers of AA chickens are significantly larger than those in CH chickens at both D4 and D7. Furthermore, this disparity is observed to increase in a time-dependent manner (**Figures 2C, D**). Collectively, these data are in support of the notion that the expression level of Atrogin-1 is a major determinant of muscle mass in broiler chickens.

### No Significant Differences in Upstream Regulators Between RJF and AA

**Figure 3:**
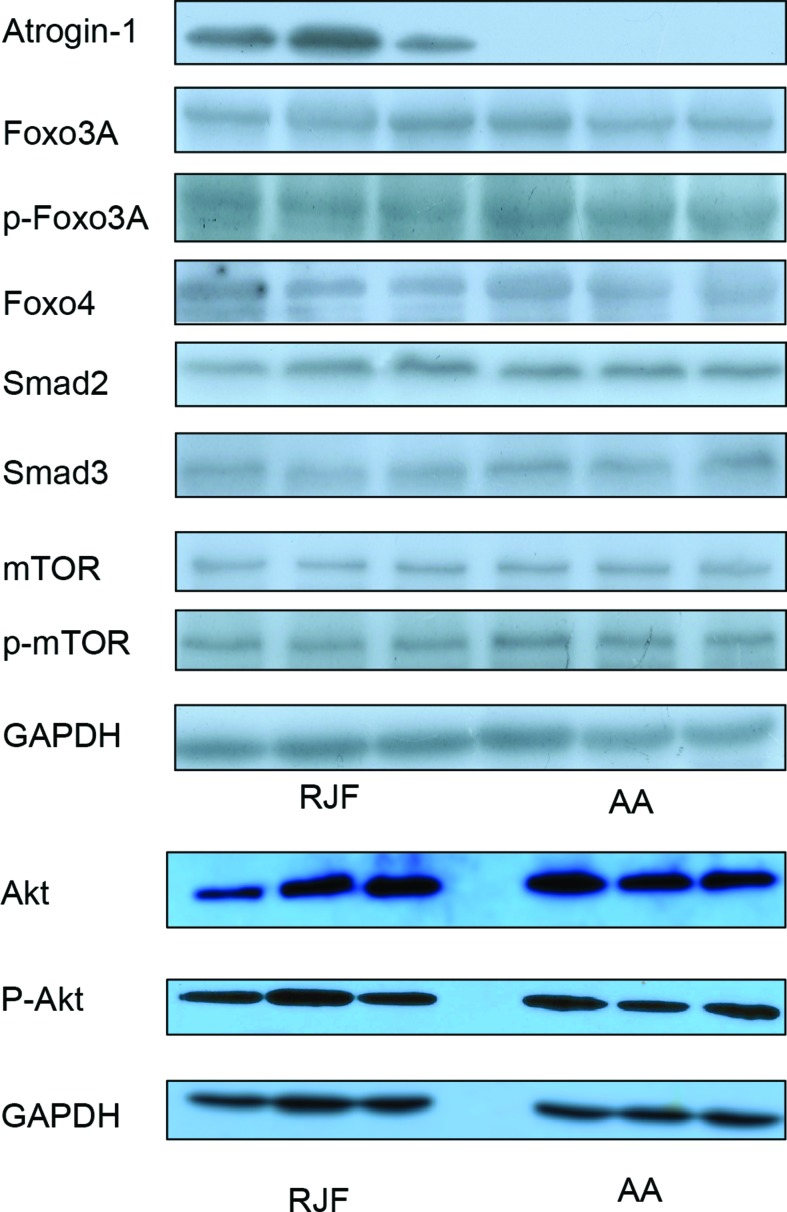
Protein levels of Atrogin-1 upstream regulators in AA and RJF chickens. Protein levels of upstream regulators of Atrogin-1 were analyzed by Western blot. Three individuals are shown in three lanes for each group. GAPDH was used as a loading control. Red Jungle Fowl (RJF) and Arbor Acres (AA).

To explore the mechanisms contributing to the discrepancies in the transcriptional regulation of Atrogin-1 in different chicken lines, we examined the protein abundances and phosphorylation levels of various upstream regulators including Akt, Foxo3, Foxo4, smad2, smad3 and mTOR. It has been demonstrated that IGF1 promotes protein synthesis *via* Akt and mTOR during muscle development (Sandri et al., 2004; Stitt et al., 2004). The transactivation of Atrogin-1 by Foxo transcription factors is known to be suppressed by the IGF-1/Akt pathway. However, we did not observe any disparities in the protein expression of these genes between the muscles of RJF and AA chickens (**Figure 3** and **Supplementary Figure 2**). It has previously been reported that TGF-β and myostatin reduced protein synthesis *via* smad2/3 and increased the expression of Foxos and Atrogin-1(Sandri, 2008; Bodine and Baehr, 2014). Therefore, we also tested the protein abundance of smad2/3, although no disparities in Smad2/3 expression between RJF and AA chickens were observed. Altogether, our data suggests that the expression level of Atrogin-1 in chicken muscles is not determined by alterations of upstream regulatory factors.

### Target Sequencing Results of the Atrogin-1 Gene Region Among Chicken Lines Show a Reduced Heterozygosity in Broiler Chickens

**Figure 4:**
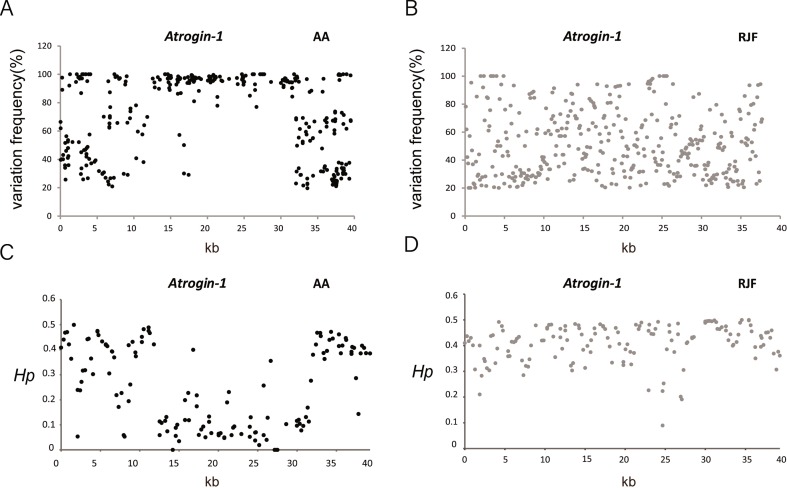
Sequencing result showed a reduced heterozygosity in Broiler chickens. **(A**–**B)** Distribution of the variation frequency for single nucleotide polymorphisms (SNPs) across the Atrogin-1 gene region in AA and RJF chickens. Variation frequency is the ratio of mutant reads to total reads for each SNP. The mutant site with the variation frequency above 20% is considered a polymorphism site by SNP calling. **(C**–**D)** Distribution of *Hp* values for all windows across the Atrogin-1 gene region in AA and RJF chickens. Heterozygosity, *Hp*, is calculated in sliding 500 bp windows with a step size of 250 bp for AA and RJF chickens. Red Jungle Fowl (RJF) and Arbor Acres (AA).

To discover SNPs of the Atrogin-1 gene region among chicken breeds, we targeted a 40 kb sequence (chr 2: 143,690,694-143,730,638 bp) including 10 kb upstream and 6 kb downstream of the Atrogin-1 gene. RJF, CH, Tibetan, Silkie, Fast Silkies, Mini chickens, Cobb and AA chicken breeds were used (N = 50) (CH, Tibetan, Silkie, Fast Silkie and Mini chickens are Chinese native chicken breeds, while Cobb and AA chickens are commercial broiler lines which are derived from Cornish crosses). The PCR products were purified and subsequently sequenced by Ion torrent. Different chicken breeds were separated by barcodes, which gave us SNP information in this region for the eight breeds. The variation frequency of all the SNPs and the distribution of the *Hp* (heterozygosity) value showed a reduced heterozygosity in AA chickens but not in RJF chickens (**Figure 4**), which may suggest a selection in this 40 kb region in broiler chickens. The variation frequency and *Hp* results of the other six breeds also proved that Cobb broiler chickens has a reduced heterozygosity in this region (**Supplementary Figure 3**). These results suggest that a mutation exist in the Atrogin-1 gene region which is selected during artificial selection for the growth traits and consequentially affects the expression of this gene.

In order to validate whether the selection of this gene region is a universal trend in broiler chickens, we included more breeds to check the mutant frequencies. According to re-sequencing results of High-Quality chicken Line A (HQLA) (a commercial broiler breed) and native LiYang (LY) broiler chickens, a similar reduced heterozygosity was found in the Atrogin-1 gene regions (**Supplementary Figure 4**).

To prove our hypothesis and to identify the functional mutation in Atrogin-1 of broiler chickens, we first obtained all the SNPs from the sequencing results. We found 46 candidate SNPs in broiler chickens, which showed a wild type in RJF and CH chickens (**Supplementary Table 3**). Finally, the eight most promising mutations were selected from the 46 SNPs based on the following criteria: the mutation frequency in broiler chickens is nearly one (>0.9), but in RJF and CH chickens it is wild type (= 0) (**Table 1**). From our results, these eight candidate SNPs are almost fixed in mutant type broiler chickens, which is in stark contrast to RJF and CH chickens.

**Table 1:** Mutation frequency of candidate single nucleotide polymorphisms (SNPs) in different chicken breeds.

chr	Ref	Mut	Position	AA	Cobb	Fast Silky	Silky	Mini	Tibetan	Chahua	RJF	Position
chr2	T	C	143699522	0.9267	0.9245	0.5051	0.3579	0.3371	0	0	0	Intron 10
chr2	G	A	143708896	0.9524	0.8033	0.569	0.5867	0.3107	0	0	0	Intron 4
chr2	A	T	143711469	0.9524	0.8512	0.7019	0.4706	0.3245	0.3717	0	0	Intron 4
chr2	C	A	143715477	0.9706	0.8714	0.6719	0.6579	0.6042	0.381	0	0	Intron 2
chr2	C	T	143715485	0.9726	0.8588	0.6	0.525	0.5441	0.3	0	0	Intron 2
chr2	T	C	143715711	0.9462	0.8347	0.5586	0.4925	0.3514	0	0	0	Intron 2
chr2	A	C	143718432	1	1	0	0	0.2857	0	0	0	Intron 1
chr2	C	A	143723139	0.9474	0.5758	0.4412	0	0	0	0	0	upstream

### Transcriptional Activity Analysis of Candidate Mutations

**Figure 5:**
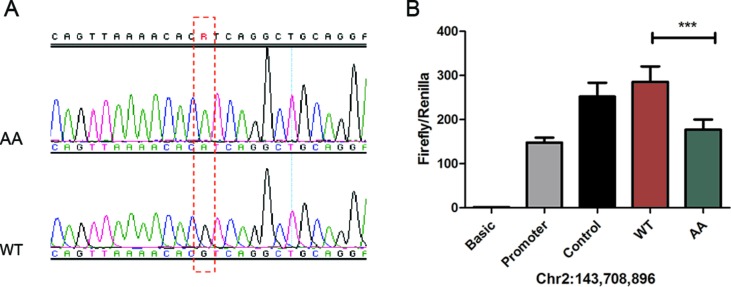
The 896G > A mutation reduced enhancer activity. **(A)** PCR sequencing result of 896G > A, RJF wild type (WT) and mutant type (AA). **(B)** The 896G > A mutation reduced enhancer activity analyzed by a luciferase reporter assay. The pGL3-Basic, pGL3-Promoter and pGL3-Control vectors were used as controls. Three technical repeats were performed for each vector in one experiment. The Firefly in relation to the Renilla luciferase level was calculated with the empty vector pGL3-Basic as a reference. The average value of three technical repeats represents one activity value. Three separate repeats were performed and used to calculate mean and SD. Wild-type (G) and mutant type (A) sequences were compared in the activity analysis using a *t*-test. *** means *p* < 0.001.

To further study the transcriptional activity of the candidate mutations, we performed luciferase reporter assays to examine the transcriptional activity of the mutant and wild type of each SNP. Considering that most of the mutations were located in introns, and not the promoter of Atrogin-1, we tested their enhancer activity. Given that mutation Chr2: 143,723,139 bp was observed to be upstream of the transcriptional start site, we also measured the promoter activity of this site. Luciferase activity was measured 24 h following transfection with mutant or wild type vectors in DF1 chicken fibroblasts (**Supplementary Figure 5**). Our results showed that there was a difference in enhancer activity between mutant-type (AA) and wild-type (RJF) of chr2: 143,708,896 bp. We observed that a G-to-A substitution led to a loss of enhancer activity in AA broiler chickens (**Figure 5**). In contrast, other mutation sites studied did not affect the transcriptional activity of Atrogin-1. This result indicates that the decreased expression of Atrogin-1 in broiler chickens could partially be ascribed to the G896A mutation; resulting in the repression of enhancer activity.

### EMSA Assays Demonstrate a Reduced Binding Activity of the Chr2:143,708,896 Loci in Broilers

**Figure 6:**
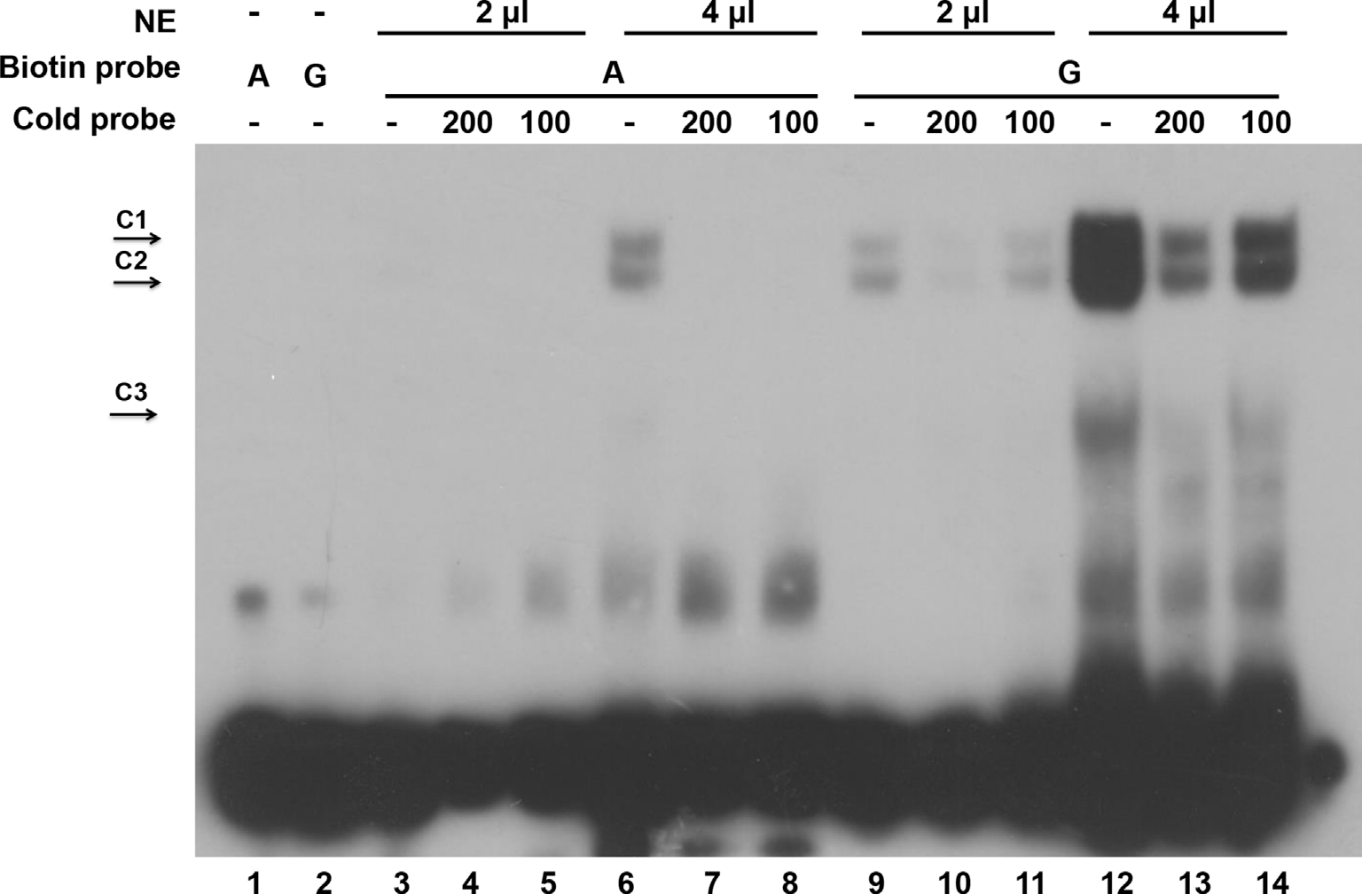
The 896G > A mutation reduced the binding of a transcription factor. Electrophoretic mobility shift assay using wild type and mutant sequences. G represents wild type probe and A represents mutant probe. NE is nuclear extract protein. C1 and C2 represent two protein complexes binding with high affinity, and C3 represents a protein complex binding with lower affinity.

To unveil the functional significance of the G896A mutation, we performed electrophoretic mobility shift assays (EMSA) using wild type (G) and mutant (A) sequences (**Figure 6**). DF-1 cell nuclear extracts were incubated with biotin-labeled wild (G) and mutant (A) double-stranded oligonucleotides. Two complexes (C1 and C2) showed higher binding ability to the wild type than the mutant probe. Binding was observed only in the wild type probe (G), but not in the mutant (A) counterpart in 2 μl of the nuclear extracts. When the volume of the nuclear extracts was increased to 4 μl, minimal binding was observed with the mutant probes (A) whereas the wild type probe (G) exhibited more profound binding compared with its counterpart in 2 μl nuclear extracts. The specificity of complex binding was confirmed by the 100- and 200-fold molar excess of unlabeled probes, which successfully compete with biotin-labeled probes. The labeled wild-type (G) and mutant-type (A) probes were specifically out competed by their own unlabeled probes. Although the unlabeled probe did not out compete the biotin-labeled probe completely, this might be due to the over-loading of nuclear extracts. Moreover, there is a specific complex C3 binding to the wild type (G) but barely to the mutant-type (A) probe.

Our result indicates that the wild-type sequence binds a transcriptional factor, and this interaction is affected by the G-to-A mutation in broiler chickens. This finding agrees with our hypothesis that the mutant allele is inhibitory to the transcriptional activity of Atrogin-1.

## Discussion

Chickens are bred all over the world and has a significant economic value as one of the major agricultural animals. Since the twentieth century, commercial chickens have been bred through a series of massive selection processes for more favorable growth traits or reproductive traits. Thus, genetic mechanisms governing chicken growth traits have immense implications not only in biological science, but also in agricultural economics.

In this study, we discovered that the expression of Atrogin-1 was inhibited in AA broiler chickens compared with RJF. As an E3 ubiquitin ligase, Atrogin-1 is considered a key factor of skeletal muscle atrophy due to its regulatory role in degradation of muscle proteins (Bodine et al., 2001; Gomes et al., 2001). The mass of skeletal muscle is determined by the balance between protein synthesis and degradation. Therefore, we speculated that the larger muscle mass in AA chickens could be in part attributed to lower levels of Atrogin-1, which would have otherwise promoted protein catabolism. Thus, we sought to unveil the mechanisms underlying the disparity of Atrogin-1 expression level among the chicken breeds. Our results show that there is a reduced heterozygosity in the Atrogin-1 gene region of broiler chickens. We postulated that the preferred allele in this gene region was selected in broiler chickens. In addition, luciferase and gel shift assays revealed a single nucleotide substitution in intron 4, which may lead to transcriptional repression of Atrogin-1 and the higher muscle weight of broiler chickens.

Measurement of Atrogin-1 expression in different embryonic (E6, E7, E11, E15 and E19) and postnatal stages (D1, D4 and D7) in CH and AA chickens revealed a difference in expression levels starting at 4 days after hatching. It is likely attributed to the more dominant anabolism than catabolism at the embryo stage. Thus, the expression of Atrogin-1 in the embryonic stage was relatively lower in both chicken breeds and showed no mRNA difference. However, after hatching the pathways related to protein degradation became more active leading to a difference in gene expression. Furthermore, the difference in muscle fiber diameter between AA and CH chickens was more profound with chicken growth. This brings forward the possibility that the expression level of Atrogin-1 would determine phenotypic variance among chicken breeds.

The transcriptional regulation of Atrogin-1 is governed by multiple signaling pathways. It is well-documented that the activation of AKT by IGF-1 provokes inhibitory phosphorylation and cytosolic retention of FOXO transcription factors; which would otherwise lead to transactivation of Atrogin-1 (Sandri et al., 2004; Stitt et al., 2004). It has also been reported that myostatin up-regulated Atrogin-1 *via* the Smad2/3 (Sandri, 2008; Bodine and Baehr, 2014). Here we did not observe any significant differences in the expression levels of the aforementioned upstream regulators between muscles of AA and RJF chickens. Our data, therefore, do not suggest the disparity in protein abundance of Atrogin-1 among the breeds as a secondary effect to aberrant catabolic signaling in the skeletal muscle.

During the process of domestication, intense efforts in the selection of preferred alleles for favorable phenotypes affect the agricultural animal genome compellingly. Strong directional selection in domestic animals leads to “selective sweeps” around the genome loci underlying selected traits (Andersson and Georges, 2004). This concept provides new insight in deciphering the genetic mechanism behind human favorable traits in animal breeding. Sutter et al. showed that the IGF1 allele is a major determinant of body size in dogs (Sutter et al., 2007). They found that there is a reduced heterozygosity in the IGF1 gene region in small (<9 kg) relative to large (>30 kg) dogs and the IGF1 single-nucleotide polymorphism haplotype is commonly found in smaller dogs. Rubin et al. used whole genome resequencing to identify selective sweeps and successfully revealed several loci under selection during chicken domestication (Rubin et al., 2010). According to our sequencing data from the eight chicken breeds, reduced heterozygosity of Atrogin-1 was only observed in broiler chickens; which may indicate that a selection in broilers where causative mutations of Atrogin-1 lead to the inhibition of gene expression. This speculation is confirmed by luciferase and gel shift assays of candidate SNPs. Our results reveal that the G to A mutation at the chr2: 143,708,896 loci of Atrogin-1 in Broiler chickens reduced the binding ability of transcriptional regulators and attenuated the enhancer activity. We predicted the potential transcription factor with PROMO (http://alggen.lsi.upc.es/cgi-bin/promo_v3/promo/promoinit.cgi?dirDB = TF_8.3) and discovered quite a lot of differences between the wild type and mutant sequence. For example, transcription factors Fos/Jun and ATF/CREB only showed up in the list of potential binding factors in wild types but not in mutants. More studies are required in terms of identification of the transcription factors that regulate gene expression.

Many mutations have been found to underlie phenotypic variations in domestic animals by QTLs or genome wide association studies (GWAS) (Andersson, 2001; Andersson and Georges, 2004; Andersson, 2009). For example, variants that modulate the expression of PLAG1 influence bovine stature (Karim et al., 2011) and muscle hypertrophy in cattle caused by deletion of MSTN (Grobet et al., 1997), and a regulatory mutation in IGF2 causes muscle growth in pigs, etc. (Van Laere et al., 2003; Jungerius et al., 2004). Compared to the use of QTL or GWAS to study genetic mechanisms of commercially important traits in domestic animals, our study found Atrogin-1 differentially expressed between chicken lines through RNA-seq and then identified a mutation in the gene region that may determine skeletal muscle development in chickens.

A spectrum of mutations is accumulated in the genome of domestic animals during the selection of preferred traits. It is worth noting that a large number of these mutations were identified in non-coding regions (Altshuler et al., 2008). Alterations of non-coding sequences were shown to have more prominent roles in rabbit domestication than those of coding sequences (Carneiro et al., 2014). In addition, muscle growth and fat deposition in pigs were found to be affected by the substitution mutation G-3,072-A in intron 3 of IGF2. Here, we have identified a single nucleotide substitution in intron 4 of Atrogin-1 which may lead to transcription repression of the gene and higher muscle mass in broiler chickens.

In summary, we found that E3 ubiquitin ligase Atrogin-1 exhibited a lower expression level in broiler chickens and that the variance expression of this gene could partly affect chicken muscle growth rate. We discovered a genetic mutation that causes reduction of Atrogin-1 expression in broiler chicken muscles. The decreased Atrogin-1 in broiler chickens would suppress the catabolism of the muscle protein and preserve muscle mass. Our study facilitates the understanding of the molecular mechanism of chicken muscle development and has a high translational impact in chicken breeding.

## Data Availability

The datasets generated for this study can be found in https://www.ncbi.nlm.nih.gov/bioproject/PRJNA530978/.

## Ethics Statement

The animal experiment was performed according to the SKLAB Animal Study Proposal (SKLAB-2012-06-02) approved by the Institutional Animal Care and Use Committee, China Agricultural University.

## Author Contributions

JL, XH and NL conceived and designed the experiments. JL, YH, QL and LL performed the experiments. JL and YW analyzed the data. JL, CF, YW, LL, YH, HL, ML, XH and NL contributed reagents, materials, and analysis tools. JL and LL wrote the paper. ML, XH and NL provided comments on the manuscript.

## Funding

This work was financially supported by the National Natural Science Foundation of China (31472083), the 948 Program of the Ministry of Agriculture of China (2012-G1[4]), the National High Technology Research and Development Program of China (2013AA102501), and the Open Research Program of State Key Laboratory for Agro-Biotechnology (2015SKLAB6-11).

## Conflict of Interest Statement

The authors declare that the research was conducted in the absence of any commercial or financial relationships that could be construed as a potential conflict of interest.

## Abbreviations

SNP, single nucleotide polymorphism; RJF, Red Jungle Fowl; AA, Arbor Acres; CH, Chahua; EMSA, electrophoretic mobility shift assay; UPS, ubiquitin–proteasome system; QTL, quantitative trait loci; GWAS, genome-wide association study; SD, standard deviation.

## Supplementary Material

Supplementary Figure 1 Relative expression level of Atrogin-1 in differential chicken species Relative expression level of Atrogin-1 was compared with the GAPDH gene. Mean ± s.d. was present for each group (n = 3). The bar means standard deviation of the three sample expressions. T-test was used to analyze the significance of every two species among RJF, CH and AA chicken. *** means P < 0.001 and ** means P < 0.01. RJF, Red Jungle Fowl; AA, Arbor Acres; CH, Chahua and Tibetan chicken.

Supplementary Figure 2 Relative protein levels of Atrogin-1 upstream regulators analyzed by imageJ The protein level for each sample was analyzed with imageJ software and normalized with its GAPDH value. The relative protein level of each gene was normalized to the corresponding gene in RJF. Mean ± s.d. was present for each group (n = 3). The bar means s.d. of the three sample. T-test was used to analyze the significance of different species. * means P < 0.05 and ns means no significant difference. RJF, Red Jungle Fowl and AA, Arbor Acres.

Supplementary Figure 3 Distribution of variation frequency and Heterozygosity of different chicken breeds

Supplementary Figure 4 Whole genome re-sequencing results of HQLA and LY broiler chicken also show a reduced heterozygosity in the Atrogin-1 gene region

Supplementary Figure 5 Effects of candidate mutations on promoter and enhancer activity WT means wild-type (Red Jungle Fowl) and AA means mutant-type (AA). Wild-type or mutant allele sequence was inserted to the pGL3-Promoter. Additionally, the wild-type or mutant allele sequence of the chr2:143,723,139 bp site was also inserted to the empty vector pGL3-Basic. -P represents promoter activity. -E represents enhancer activity. The pGL3-Basic, pGL3-Promoter and pGL3-Control vectors were used as controls. Three technical repeats were performed for each vector in one experiment. Firefly in relation to Renilla luciferase levels was calculated with the empty vector pGL3-Basic as reference. The average value of three technical repeats represents as one activity value. Three separate repeats were performed and used to calculate mean and standard deviation (SD). Wild-type and mutant type sequences were compared in the activity analysis using t-test. *** means p < 0.001. (A) Chr2: 143,699,522 bp (B) Chr2: 143,708,896 bp (C) Chr2: 143,711,469 bp (D) Chr2: 143,715,477 bp (E) Chr2: 143,715,485 bp (F) Chr2: 143,715,711 bp (G) Chr2: 143,718,432 bp (H) Chr2: 143,723,139 bp.
