# Supplementary figures and images for: A Discovery of a Genetic Mutation Causing Reduction of Atrogin-1 Expression in Broiler Chicken Muscle
Source: Front Genet. 2019 Aug 15;10:716. doi: 10.3389/fgene.2019.00716 (PMC6704234; doi:10.3389/fgene.2019.00716)

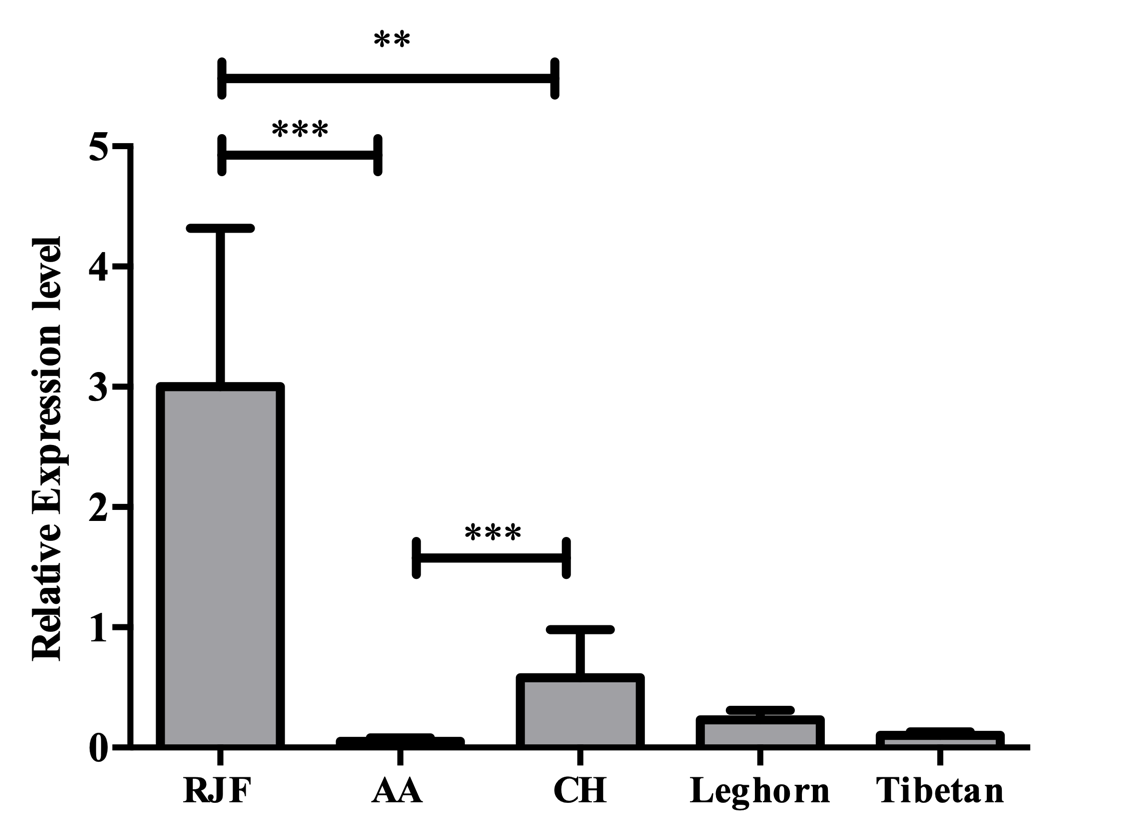

Supplement: Supplementary Figure 1 — Relative expression level of Atrogin-1 in differential chicken species Relative expression level of Atrogin-1 was compared with the GAPDH gene. Mean ± s.d. was present for each group (n = 3). The bar means standard deviation of the three sample expressions. T-test was used to analyze the significance of every two species among RJF, CH and AA chicken. *** means P < 0.001 and ** means P < 0.01. RJF, Red Jungle Fowl; AA, Arbor Acres; CH, Chahua and Tibetan chicken. [file Image_1.tiff]

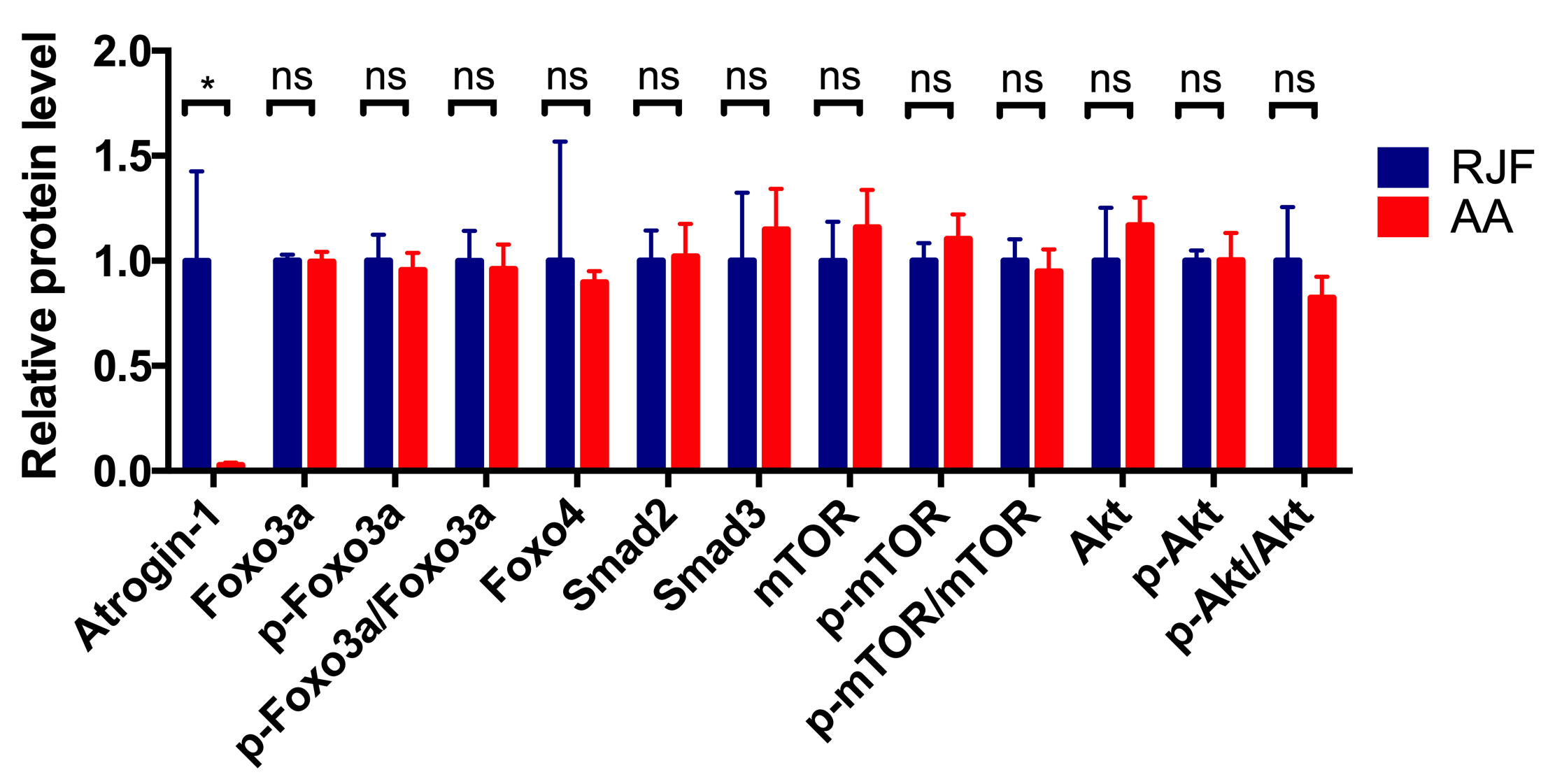

Supplement: Supplementary Figure 2 — Relative protein levels of Atrogin-1 upstream regulators analyzed by imageJ The protein level for each sample was analyzed with imageJ software and normalized with its GAPDH value. The relative protein level of each gene was normalized to the corresponding gene in RJF. Mean ± s.d. was present for each group (n = 3). The bar means s.d. of the three sample. T-test was used to analyze the significance of different species. * means P < 0.05 and ns means no significant difference. RJF, Red Jungle Fowl and AA, Arbor Acres. [file Image_2.tiff]

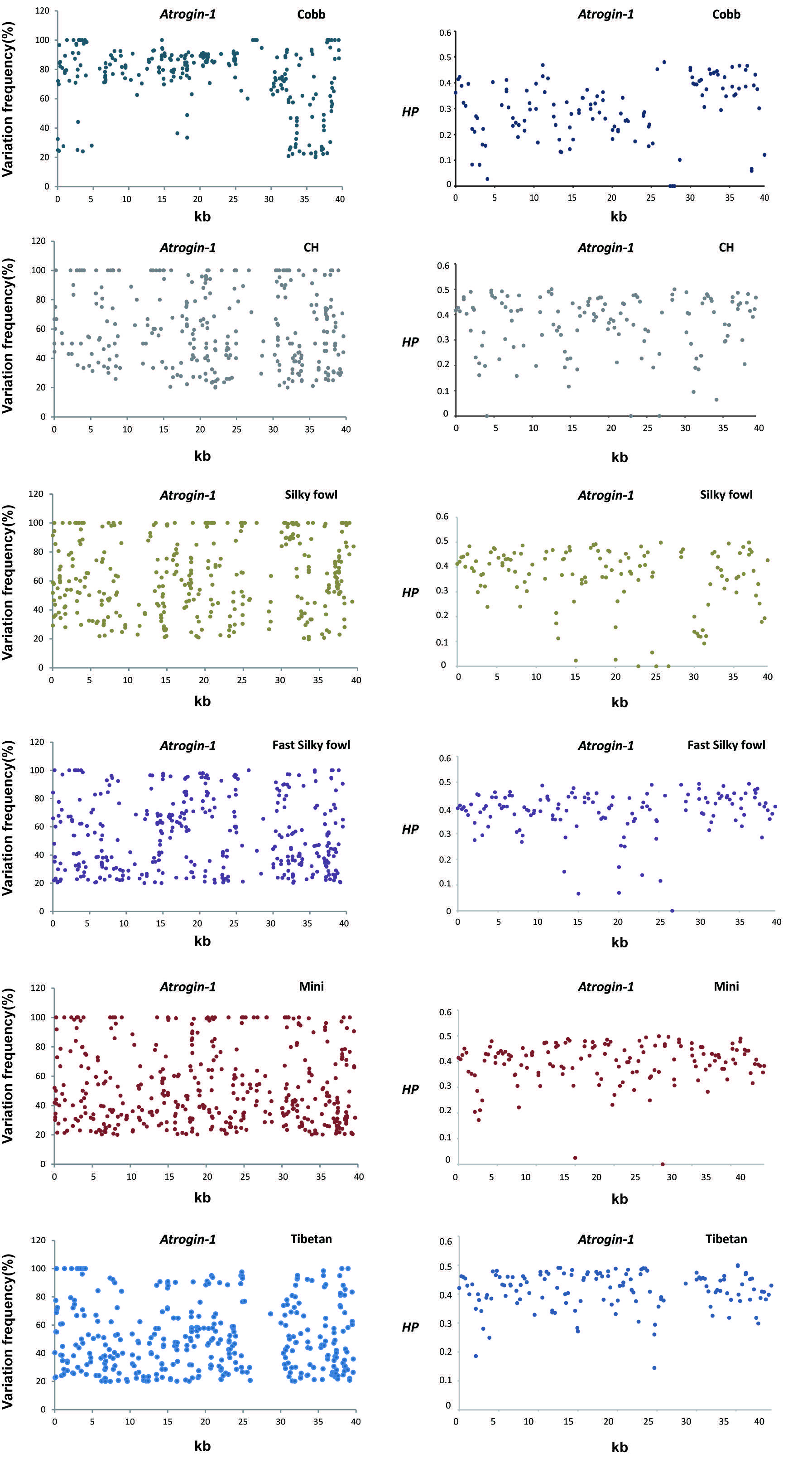

Supplement: Supplementary Figure 3 — Distribution of variation frequency and Heterozygosity of different chicken breeds [file Image_3.tif]

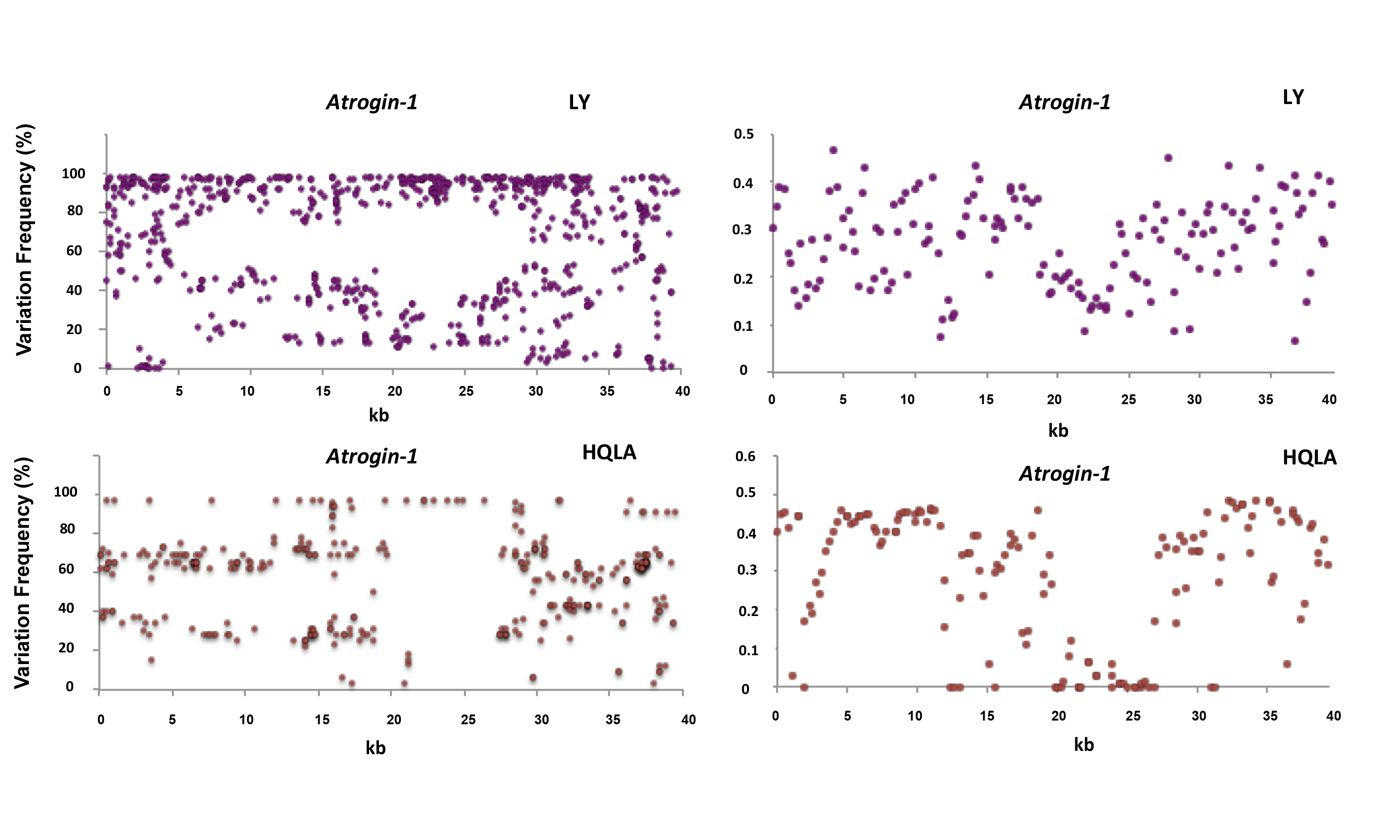

Supplement: Supplementary Figure 4 — Whole genome re-sequencing results of HQLA and LY broiler chicken also show a reduced heterozygosity in the Atrogin-1 gene region [file Image_4.tif]

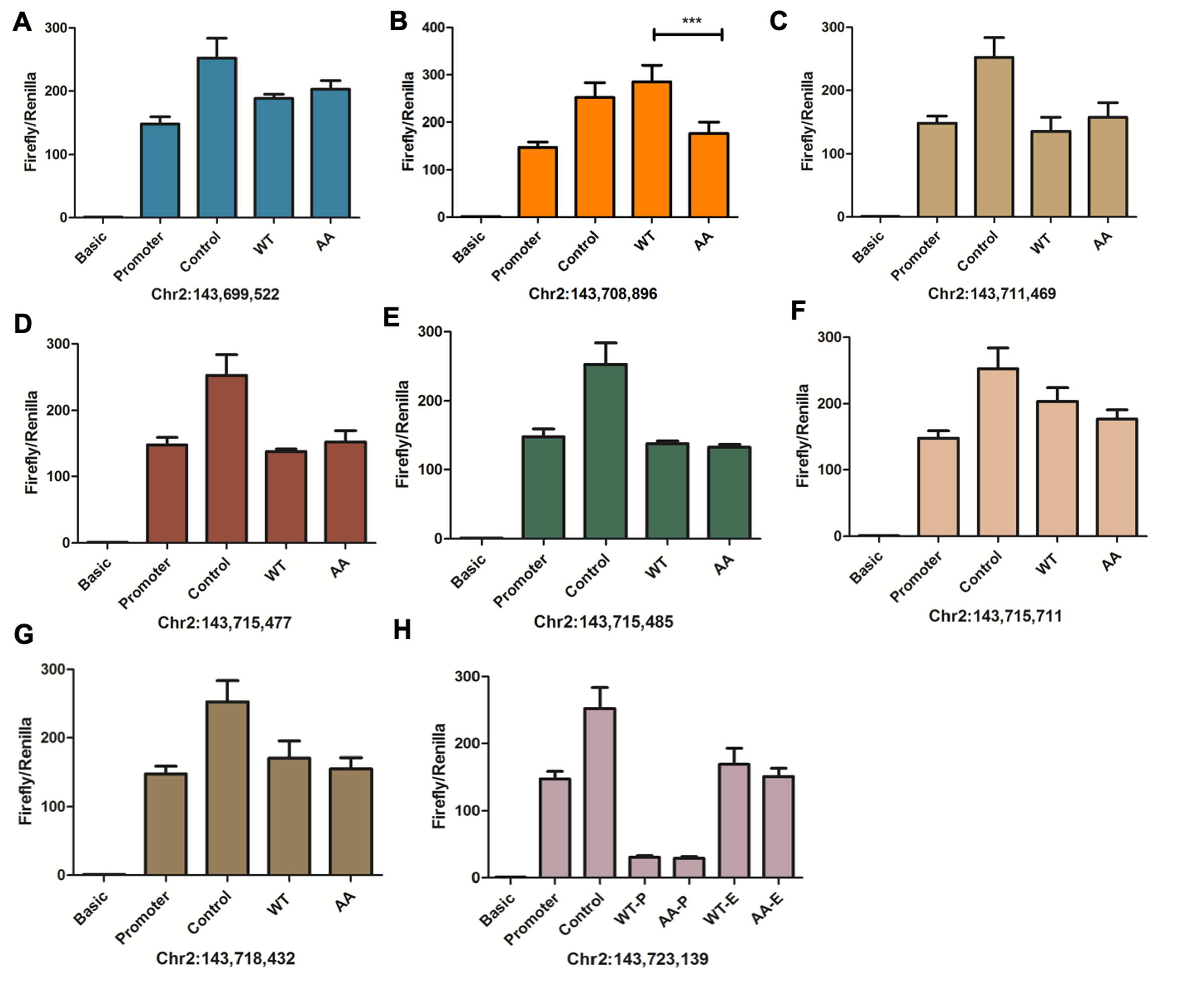

Supplement: Supplementary Figure 5 — Effects of candidate mutations on promoter and enhancer activity WT means wild-type (Red Jungle Fowl) and AA means mutant-type (AA). Wild-type or mutant allele sequence was inserted to the pGL3-Promoter. Additionally, the wild-type or mutant allele sequence of the chr2:143,723,139 bp site was also inserted to the empty vector pGL3-Basic. -P represents promoter activity. -E represents enhancer activity. The pGL3-Basic, pGL3-Promoter and pGL3-Control vectors were used as controls. Three technical repeats were performed for each vector in one experiment. Firefly in relation to Renilla luciferase levels was calculated with the empty vector pGL3-Basic as reference. The average value of three technical repeats represents as one activity value. Three separate repeats were performed and used to calculate mean and standard deviation (SD). Wild-type and mutant type sequences were compared in the activity analysis using t-test. *** means p < 0.001. (A) Chr2: 143,699,522 bp (B) Chr2: 143,708,896 bp (C) Chr2: 143,711,469 bp (D) Chr2: 143,715,477 bp (E) Chr2: 143,715,485 bp (F) Chr2: 143,715,711 bp (G) Chr2: 143,718,432 bp (H) Chr2: 143,723,139 bp. [file Image_5.tif]
